# Appearance and recurrence of odontogenic keratocysts

**DOI:** 10.1002/cre2.796

**Published:** 2023-10-05

**Authors:** Jaakko Pylkkö, Jaana Willberg, Auli Suominen, Hanna K. Laine, Jaana Rautava

**Affiliations:** ^1^ Department of Oral Pathology and Oral Radiology, Institute of Dentistry, Faculty of Medicine University of Turku Turku Finland; ^2^ Department of Pathology Turku University Central Hospital Turku Finland; ^3^ Department of Community Dentistry University of Turku Turku Finland; ^4^ Department of Oral and Maxillofacial Diseases, Clinicum, Faculty of Medicine University of Helsinki Helsinki Finland; ^5^ Department of Pathology, HUS Diagnostic Center, HUSLAB Helsinki University Hospital Helsinki Finland

**Keywords:** histological appearance, odontogenic keratocyst, recurrence, satellite cyst

## Abstract

**Objective:**

The purpose of this study was to evaluate the appearance, histopathological features, and recurrence of odontogenic keratocysts (OKCs) from a large single institute registry over a 36‐year period.

**Materials and Methods:**

A total of 226 cases of OKC were identified in 174 patients over a 36‐year period in a single institute in Southwestern Finland. Histological specimens were re‐evaluated. The patient's age, sex, location, recurrence, and histopathological features of the OKC were the study variables.

**Results:**

OKCs occurred more frequently in men, the mean age was 46 years, and the most frequent site was the lower jaw. Recurrence rate was 21%. Histopathologically, inflammation was present in 95% and satellite cysts in 10% of cases. In patients diagnosed with satellite cysts, OKC recurred in 50% of cases, while the corresponding figure for patients without satellite cysts was 17%.

**Conclusions:**

Compared with the literature, patients were older and inflamed cysts were found more frequently. Satellite cysts occurred only in association with chronic inflammation. Based on the results, regular radiographic evaluation is important among patients aged 10–29 years to detect OKCs and to treat them before enlargement, infection, and inflammation. Satellite cysts should be reported and may be a sign of increased risk of OKC recurrence.

## 1 INTRODUCTION

Odontogenic keratocyst (OKC) represents 5%–15% of all odontogenic cysts (Odell, 2017). OKC has a slight male predilection (1.5:1), and the peak incidence is in the third decade of life (Odell, 2017). Occurrence is more common in the posterior region of the dental arch, the angle, or the ramus of the mandible (66%–77%) than in the maxilla (Ortega García de Amezaga et al., 2008; Regezi et al., 2016; Scully, 2013).

OKC purportedly arises from remnants of the dental lamina. As a definition of a cyst, OKC is an epithelium‐lined pathologic space filled with fluid or occasionally semisolid material with shedding keratin. Histologically, the epithelial lining is parakeratinized, typically 6–10 cells thick, uniform, and an even or a varyingly corrugated strand of epithelium. The basal layer of the cyst epithelium shows a palisaded pattern with polarized, intensely stained nuclei. Epithelial detachment from the connective tissue is fairly common. Occasionally encountered features include the budding of the epithelia to the underlying connective tissue and the formation of satellite cysts (also called daughter cysts) (Regezi et al., 2016). Most OKCs are asymptomatic until the bone is expanded or they become infected (Odell, 2017; Scully, 2013). Dense and/or intense inflammation often results in loss of histological characteristics and may challenge the histopathological diagnosis.

OKC appears either as a solitary lesion or multiple lesions. Multiple OKCs are known to have a strong association with Gorlin‐Goltz syndrome (also known as nevoid basal cell carcinoma syndrome [NBCCS]). Syndromic OKCs may have a mutation in protein patched homolog 1 (*PTCH*). However, mutations in the same gene have been often found in sporadic OKCs (Manfredi et al., 2004; Ortega García de Amezaga et al., 2008). In short, a nonfunctioning *PTCH* leads to an overexpression of SHH (Sonic Hedgehog) and/or SMO (Smoothened protein) causing an increased cell proliferation.

OKCs typically show rapid, aggressive growth and high rates of recurrence of 15%–63% (Bande et al., 2010; Chrcanovic & Gomez, 2017; Fidele et al., 2019). Patients with NBCCS show recurrence rates of 25%–52% (Tarakji et al., 2013). Treatment is traditionally surgical excision and management of the bone surrounding the cyst by mechanical or chemical means. Treatment of choice may have a large impact on the recurrence rate. For enucleation and curettage, recurrence has been shown in 63% of cases, while enucleation and Carnoy's solution (as low as 2.5%) can markedly lower the likelihood of recurrence (Abdullah, 2011). OKC may show satellite cysts whose prevalence varies between 20% and 32% in nonsyndromic cases and between 56% and 71% in syndromic cases. Inadequate removal of satellite cysts leaves behind debris with growth potential that may produce a recurring lesion (Pogrel, 2013).

The aim of this study was to evaluate the appearance, histopathological features, and recurrence of OKCs from a large single institute registry over a 36‐year period.

## 2 MATERIALS AND METHODS

### 2.1 Patient and sample material

This study was approved by the Institute of Dentistry, University of Turku, Turku, Finland, as a registry holder. All histopathological material of OKCs diagnosed in 1980–2016 was gathered from the pathology archives of the Institute of Dentistry, University of Turku, without patient identification. The inclusion criteria were as follows: (1) histological slides and referral letter were available and (2) histopathological diagnosis of OKC was confirmed by re‐evaluation. For the histopathological analysis, if the patient had multiple OKC samples during the period the first sample was chosen for each patient. Recurrent OKCs were registered if the same patient had multiple OKC samples over the study period. The patient's age, gender, and the location of OKCs were collected from pathological‐anatomical‐diagnosis (PAD) referral letters. Not all referral letters included all information (age, gender, and location). Data consisted exclusively of Finnish citizens with Finnish social security number.

### 2.2 Histopathology

OKC histopathology was re‐evaluated by two authors (J. P. and J. R.). J. P. evaluated all cases and 26 cases (9.9%) were randomly chosen to represent a quality control conducted by an oral pathologist (J. R.). The histopathological slides were analyzed according to World Health Organization (WHO) 2017 guidelines (EI‐Naggar et al., 2017). The following characteristics were recorded for each specimen to fulfill inclusion criteria: (1) palisading of the basal lamina (yes/no), (2) parakeratinization of the odontogenic epithelia (yes/no), and in addition (3) thickness of the epithelia (even or varying), (4) detachment of the epithelia from the connective tissue (yes/no), (5) presence of satellite cysts (yes/no), and (6) dense/intense inflammation focal/generalized (yes/no).

Satellite cysts were analyzed by evaluating consecutive tissue sections of the samples and determining whether sporadic satellite cysts are connected to the main lumen or not.

### 2.3 Statistical analysis

All statistical analyses were performed with SPSS Statistics version 25. Mean and standard deviation were calculated for continuous age and proportions for categorical sex. The location of OKCs and the distributions of variables were analyzed. When appropriate, normality was evaluated by the Kolmogorov–Smirnov test. The association between inflammation and satellite cysts was analyzed using Binomial Exact. *χ* ^2^ and Fisher's exact tests (two‐sided) were used to analyze correlations between gender, location, and histological characteristics. Fisher's exact test was used to compare recurrent satellite cyst cases. The limit of statistical significance was set at *p* < .05.

## 3 RESULTS

### 3.1 Samples and patients

Over the 36‐year period, 28,940 biopsies were diagnosed at the Institute of Dentistry, University of Turku, Finland. In total, 378 samples (1.3%) were diagnosed as OKC (formerly, keratocystic odontogenic tumor) covering also recurrent OKC samples. The first OKC sample of each patient was evaluated resulting 226 OKC cases from 174 patients. Of the patients, 56.9% were men (99/174) and 43.1% women (75/174). Patients' mean age at first OKC diagnosis was 46 years (range: 9–89 years, median: 48 years; data missing for one patient). The peak incidences were 19–27 years (18.5%) for men and 59–65 years (17.3%) for women. Age distribution did not follow normal distribution (one‐sample Kolmogorov–Smirnov test; *p* = .003). Interestingly, nine (6.2%) patients were under 18 years (mean age 15 years); five of them were boys and four were girls. In this group, the recurrence rate was 18.2% due to two Gorlin‐Goltz syndrome patients with recurrent OKCs.

Information on OKC location was available for 165 cases (94.8%). Most OKCs (76.5%) were located in the mandible.

### 3.2 OKCs and histopathology

**Figure 1:**
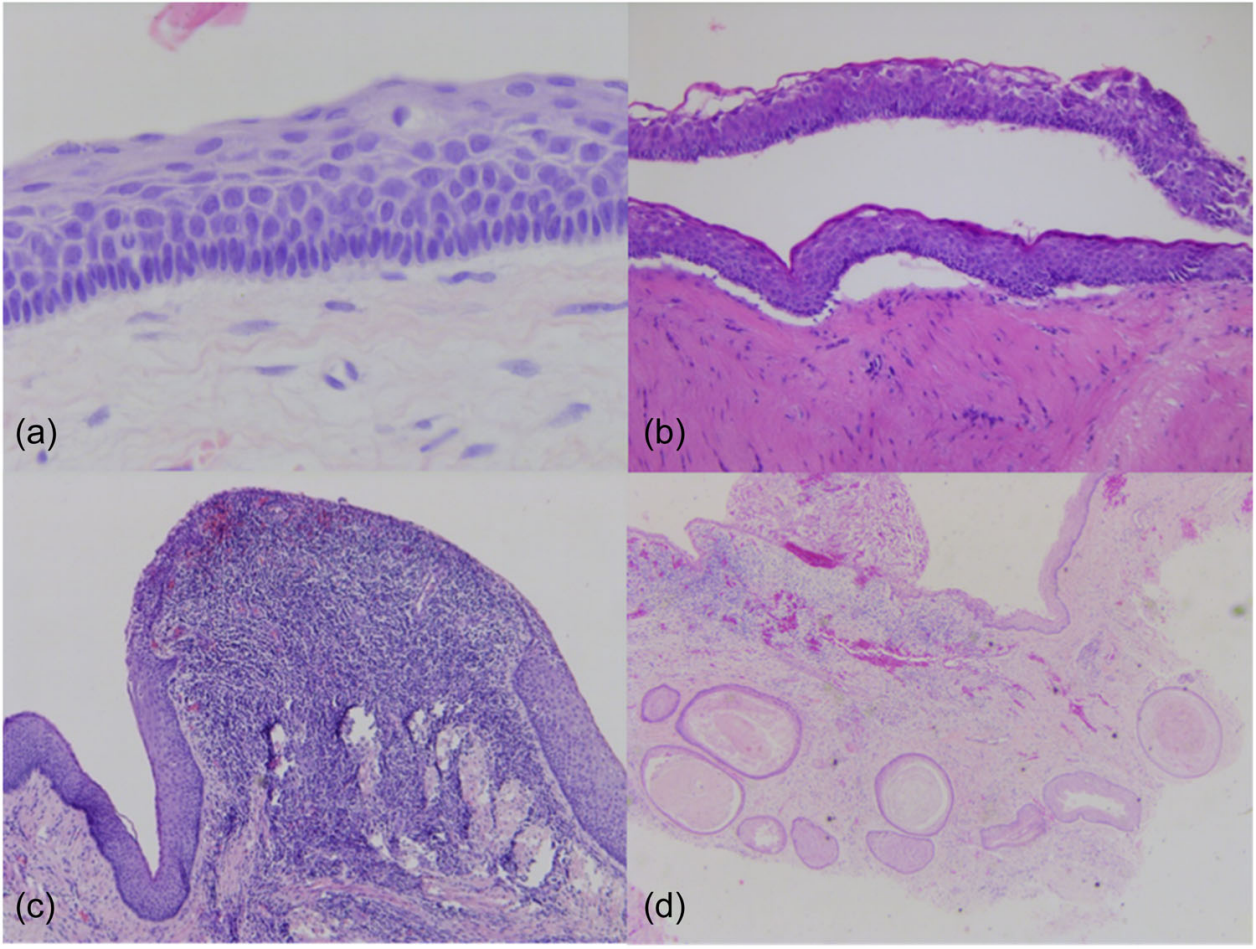
Histological appearance of odontogenic keratocysts. (a) Palisadation and parakeratinization. (b) Even epithelia and detachment from connective tissue. (c) Chronic inflammation in underlying connective tissue is destroying characteristic features of cyst epithelium. (d) Satellite cysts.

As inclusion criteria, all OKCs presented parakeratinized cyst epithelium and palisaded basal cells (Figure 1a). Thickness of the epithelium was even in 97.1% (169/174) of cases. Of the five OKCs with variable thickness of the cyst‐epithelium, two were located in the mandible, one in the maxilla, and two were unknown. Cyst‐epithelium detachment from the connective tissue was present in 64.6% (113/174) of cases (Figure 1b). Detachment was somewhat more common in the mandible (84/126, 66.7%) than in the maxilla (22/38, 57.9%). Cyst‐epithelium detachment appeared more often in males (68.7%) than females (58.3%). Inflammation was present to some degree in 165/174 cases (94.8%) (Figure 1c). Inflammation was chronic (98.8%) in all but two cases. Satellite cysts were present in 17 cases (9.8%) (Figure 1d). Satellite cysts were slightly more common among women than men (12.0% vs. 8.1%). Satellite cyst appearance was more common in the maxilla (5/38, 13.2%) than in the mandible (9/126, 7.1%).

### 3.3 Follow‐up

In 37 patients, 174 OKCs (21.3%) recurred from one to five times (Table 1). Of the 150 cases without satellite cysts, 25 (16.7%) had a recurrence of up to three times. In cases with satellite cysts, 12/24 (50%) recurred up to five times. There was no correlation between histological characteristics and age, sex, or location. Satellite cysts were present only with inflammation (*p* = .001) (Table 2). Epithelium with varying thickness did not show any detachment from the connective tissue (*p* = .005). Patients with satellite cysts showed a significantly higher rate of recurrences (50%, 12/24) than patients without satellite cysts (16.7%, 25/150) (*p* = .001) (Table 1).

**Table 1:** Presence of satellite cysts in recurrent and nonrecurrent parakeratinized odontogenic keratocysts.

Recurrences	Appearance of satellite cysts		**Total**
	Not present	Present	
0	125	12	137
1	20	7	27
2	4	3	7
3	1	1	2
5	0	1	1
**Total**	150	24	174

**Table 2:** Presence of inflammation and satellite cysts in parakeratinized odontogenic keratocysts.

Inflammation	Satellite cysts	No satellite cysts	Total
None	0	9	9
Chronic	17	146	163
Acute	0	2	2
**Total**	17	157	174

## 4 DISCUSSION

To evaluate the histopathological features of OKCs, we reviewed retrospectively 174 OKC patients diagnosed between the years 1980 and 2016 at the Institute of Dentistry, University of Turku, Turku, Finland. According to our results, OKC patients in Southwestern Finland were older, the cysts were more commonly inflamed, and satellite cysts were exclusively present with chronic inflammation.

According to the literature, OKC occurs more commonly in males (1.2–1.5:1) (Myoung et al., 2001; Neville et al., 2009; Odell, 2017; Thompson et al., 2016) and in the mandible (75%–77.0%) (Deepthi et al., 2016; Myoung et al., 2001; Neville et al., 2009). Similarly in the current study, OKCs showed a male predilection and the mandible was the most common location. The incidence of OKC has been rarely reported. In the current study, the incidence was 7.7 cases per year in Southwestern Finland, which is an area covering 9% of the Finnish population.

Interestingly, patients in our study were older (mean age 46 years) at the time of the OKC diagnosis than previously described in the literature (Odell, 2017). In our study, older age at OKC diagnosis could reflect a diagnosis delay. Since OKC has a tendency for being asymptomatic, these cysts could persist for years before the discovery due to symptoms like swelling or infection. Hypothetically in our study, the delay of the diagnosis or treatment could have given time for the immune system to react to cyst formation escalating inflammation around OKC. As it was a registry‐based study setup, we were unable to rule out reasons such as extractions and/or biopsies. Previously inflammation has been described in 76% of OKCs (Kaplan & Hirshberg, 2004; Rodu et al., 1987). Inflammation was present in 95% of our cases, supporting the idea of a late diagnosis with larger and infected OKCs. Inflammation was considered present if generalized or focal dense/intense inflammation was found in any section of the sample.

In our study, 21% of patients had recurrence. The reasons for the high recurrence rate of OKC are not known. Treatment methods may have an impact on the recurrence rate, for example, enucleation of OKC and handling with Carnoy's solution could lower the likelihood of recurrence (Abdullah, 2011). In addition, the thin, fragile epithelial cyst lining of OKC can easily break during enucleation, leading to inadequate removal and recurrences. Another reason could be microscopic satellite cyst formation. In our study, there was a slightly lower occurrence of satellite cysts (10%) than described earlier (20%–30%) (Myoung et al., 2001; Pavelić et al., 2014). Myoung et al. (2001) demonstrated that satellite cysts might affect recurrence, which was consistent with our findings. Nonetheless, there are other studies that have not found any association between OKC satellite cyst and recurrence rate (Kaczmarzyk et al., 2018; Kuroyanagi et al., 2009; Naruse et al., 2017). Moreover, in our study, satellite cysts of OKC were present only with chronic inflammation and the incidence was slightly higher in the maxilla. More research is warranted to clarify the reasons for recurrences and the relationship between satellite cysts and OKC recurrence.

Syndromic OKCs more commonly appear in the first three decades of life, but for nonsyndromic OKCs the range is wider (Borghesi et al., 2018; Brannon, 1976; Woolgar et al., 1987). In our study, 6% of the patients were under 18 years. Among pediatric patients, the recurrence rate was 18%, which was similar to the whole cohort (21%). Two patients with recurrent OKCs had Gorlin‐Goltz syndrome.

The strength of our study is the large sample size and long follow‐up time. Limitations include the use of PAD referral letters instead of patient medical records. However, age, sex, and location as well as recurrences are reliably recorded in the PAD referral letters and pathological files. In the future, more detailed clinical and radiological information on OKC from patient medical records will be helpful.

## 5 CONCLUSIONS

OKC patients in Southwestern Finland were older and OKCs were more commonly inflamed compared to the current literature. Our results suggest that inflammation might induce the occurrence of satellite cysts. This emphasizes the importance of regular radiographic examinations, such as panoramic X‐rays, in the second and third decades of life. Histopathologically, the appearance of satellite cysts in the OKC should be reported and may be a sign of an increased risk of OKC recurrence.

## AUTHOR CONTRIBUTIONS

**Jaakko Pylkkö**: Methodology; formal analysis; investigation; writing—original draft. **Jaana Willberg**: Methodology; investigation; writing—review and editing; supervision. **Hanna K. Laine**: Methodology; writing—review and editing. **Auli Suominen**: Methodology; formal analysis. **Jaana Rautava**: Methodology; investigation; writing—review and editing; supervision; project administration; resources.

## CONFLICT OF INTEREST STATEMENT

The authors declare no conflict of interest.

## Data Availability

The data supporting the findings of this study are available from the corresponding author upon reasonable request.
